# High throughput sequencing unravels tomato-pathogen interactions towards a sustainable plant breeding

**DOI:** 10.1038/s41438-021-00607-x

**Published:** 2021-08-01

**Authors:** Maria Doroteia Campos, Maria do Rosário Félix, Mariana Patanita, Patrick Materatski, Carla Varanda

**Affiliations:** 1 MED – Mediterranean Institute for Agriculture, Environment and Development, Instituto de Investigação e Formação Avançada, Universidade de Évora, Pólo da Mitra, Ap. 94, 7006-554 Évora, Portugal; 2 MED – Mediterranean Institute for Agriculture, Environment and Development & Departamento de Fitotecnia, Escola de Ciências e Tecnologia, Universidade de Évora, Pólo da Mitra, Ap. 94, 7006-554 Évora, Portugal

**Keywords:** High-throughput screening, Plant breeding

## Abstract

Tomato (*Solanum lycopersicum*) is one of the most economically important vegetables throughout the world. It is one of the best studied cultivated dicotyledonous plants, often used as a model system for plant research into classical genetics, cytogenetics, molecular genetics, and molecular biology. Tomato plants are affected by different pathogens such as viruses, viroids, fungi, oomycetes, bacteria, and nematodes, that reduce yield and affect product quality. The study of tomato as a plant-pathogen system helps to accelerate the discovery and understanding of the molecular mechanisms underlying disease resistance and offers the opportunity of improving the yield and quality of their edible products. The use of functional genomics has contributed to this purpose through both traditional and recently developed techniques, that allow the identification of plant key functional genes in susceptible and resistant responses, and the understanding of the molecular basis of compatible interactions during pathogen attack. Next-generation sequencing technologies (NGS), which produce massive quantities of sequencing data, have greatly accelerated research in biological sciences and offer great opportunities to better understand the molecular networks of plant–pathogen interactions. In this review, we summarize important research that used high-throughput RNA-seq technology to obtain transcriptome changes in tomato plants in response to a wide range of pathogens such as viruses, fungi, bacteria, oomycetes, and nematodes. These findings will facilitate genetic engineering efforts to incorporate new sources of resistance in tomato for protection against pathogens and are of major importance for sustainable plant-disease management, namely the ones relying on the plant’s innate immune mechanisms in view of plant breeding.

## Introduction

Plant–pathogen interaction triggers the activation of signals that result in rapid defense response against an array of plant pathogens. The responses of plants to biotic stresses are very complex as the multitude of interactions involves at least two organisms, the plant and the pathogen. Plants have evolved a complex defense system against pathogens including cascade signaling activation, the regulation of gene expression, synthesis of defensive metabolites as well as hormone balancing, that prevents or hinders colonization by most potential pathogens^1^.

Investigation into the molecular basis of pathogen resistance reveals a suite of cellular receptors that performs direct detection of pathogenic molecules. The first stage of defense in plants is triggered by pattern recognition receptors (PRRs) that recognize pathogen-associated molecular patterns (PAMPs) inducing PAMP-triggered immunity (PTI), preventing pathogen colonization^2–4^. PRRs also detect wall-associated kinases (WAKs) that detect damage-associated molecular patterns (DAMPs) resulting from cellular damage during infection^5,6^. PTI leads to the production of reactive oxygen species (ROS), activation of mitogen-activated protein kinase (MAPK) cascades, G-proteins, ubiquitin, calcium, hormones, transcription factors (TFs), and epigenetic modifications that regulate the expression of pathogenesis-related (PR) genes^7–11^. In order to prevent further infections, plants develop several responses such as hypersensitive response (HR), cell wall modification, closure of stomata, or the production of various anti-pathogen proteins and compounds (e.g., chitinases, protease inhibitors, defensins, and phytoalexins)^12,13^. HR is one of the most commonly used immune responses, causing planned cell death in the area surrounding an infection. This establishes a quarantine zone to stop the pathogen from spreading, an effective technique against pathogens that require living tissue (biotrophs)^14^.

Pathogens have evolved to acquire effector molecules to counteract the plant PTI mechanism and ensure pathogenicity. This prompted plants in turn to develop intracellular receptors (R proteins) that recognize the pathogen/pest effector(s) and initiate an additional level of defense termed effector-triggered immunity (ETI)^15,16^. Receptors with nucleotide-binding domains and leucine-rich repeats (NBS-LRRs, also known as NLR immune receptors) are the most abundant plant disease resistance (R) genes, and detect effectors that pathogens use to facilitate infection^17^. This interaction is also referred to as incompatible interaction and is generally characterized by a vast transcriptional reprogramming after recognition of the pathogen/pest effector molecule(s)^18^.

New insights into the plant immune system can be achieved through genomic approaches^16,19^. The identification of plant key functional genes in susceptible responses and the understanding of the molecular basis of compatible interactions are possible with techniques that allow the study of differential gene expression. Although various resistance genes have been functionally identified in the disease-resistance system, there is still a lot to know about the complex molecular mechanisms involved in defense responses.

Tomato (*Solanum lycopersicum*) is one of the most economically important vegetables throughout the world; it is a member of the genus *Solanum* within the family Solanaceae, which includes several other commercially important species. It is estimated that 4.6 million hectares of tomato are grown worldwide annually producing more than 126 million metric tonnes (http://faostat.fao.org). Tomato can be grown in a variety of geographical zones, in open fields or greenhouses, and the fruit can be harvested either manually or mechanically. Its fruits are end products both for the fresh market and the food processing industry. Tomato plants are affected by different pathogens that cause symptoms including wilts, leaf spots/blights, fruit spots, and rots that consequently reduce yield and affect product quality^20^. Therefore, tomato arises as an important culture for the implementation of high-throughput methods that enable wide transcriptome profiling and the identification of differential expression genes (DEGs) in response to pathogens. The information generated by large-scale genome sequencing is leading to a major revolution in the understanding of tomato biology.

In this review we point to the relevance of tomato as a model plant, focusing on its importance to study biotic stress and plant-pathogen interaction. We summarize the current knowledge gathered from the use of RNA-seq technology to obtain transcriptome changes in tomato plants in response to important pathogens, contributing for the identification of tomato regulatory components involved in protection against pathogens, in view of plant breeding.

## Tomato a ‘traditional’ model system

Tomato is one of the best-studied cultivated dicotyledonous plants and has been often used as a model system for plant research into classical genetics, cytogenetics, molecular genetics, and molecular biology^21^. It has been used for research into gene characterisation^22^ and gene transfer approaches^23,24^. Tomato has been useful to study other plant traits such as fruit ripening, hormone function^25^, and vitamin biosynthesis^26^. Because of its specific biochemical and molecular properties and nutritional importance, tomato is an established model to study fruit growth and development^27^. Tomato also has numerous mapped traits, developed DNA markers, abundant collections of germplasm and mutants, and an increasing number of expressed sequence tags (ESTs)^28–32^. There are several characteristics that make tomato an ideal model organism for both basic and applied research programs. A high-quality genome sequence for tomato became available in 2012^33^, further enhancing the use of this species as a model to study plant defensive mechanisms. It has a diploid genome with 12 chromosome pairs and a relatively small genome size predicted in 900 Mb, with approximately 35,000 predicted protein-coding genes^34–36^. Besides that, it grows under different cultivation conditions, its life cycle is relatively short (90–120 days), it has seed production ability, and a high self-fertility and homozygosity and it have the ability of asexual propagation^37,38^.

## Why is tomato interesting to study plant defense against biotic stresses?

Tomato is affected by an abundance of diseases that reduce yield and affect product quality and, in contrast to other model organisms, it has many interesting features, as the production of fleshy fruits that are important for the human diet^37^. During cultivation or in post-harvest storage, it is susceptible to more than 200 diseases caused by an array of pathogens^39^. The diseases are mainly caused by fungi, but also by oomycetes, bacteria, viruses, viroids, and multiple nematodes (see in^20^). This large diversity of pathogens emphasizes the importance of the tomato-pathosystem as a favourable model for studying plant-pathogen interactions, contrarily to other model plants often used such as *Arabidopsis thaliana*^40–42^.

Reduction of genetic diversity among crop varieties poses risks for cultivation, especially when most varieties carry the same genetic basis for resistance to diseases and pests^43^. As a result of the reduction of genetic diversity over millennia, beneficial traits of wild species, such as disease resistance and stress tolerance, have been lost^44^. Wild relatives of tomato provide a source of valuable traits, which can be introgressed into a cultivated tomato. In this sense, natural resistance to pathogens has proven to be useful in the identification of novel immune-related genes^45,46^. As referred above, plant resistance to pathogens relies on the recognition of specific pathogen effector molecules by host plant resistance (R) proteins^16,47^. A repertoire of genetically diverse wild tomato species represents a rich source of R-genes known to be involved in tomato-pathogen recognition^41^.

We can find in the literature several examples of tomato R-genes that confer resistance to a broad number of diseases caused by several pathogenic agents. The first resistance source to *Tomato spotted wilt virus* (TSWV) was found in the wild relative *Solanum pimpinellifolium*. Seven TSWV resistance loci have been identified, designated as the dominant and allelic *Sw-1a* and *Sw-1b*, three recessive genes *sw-2, sw-3*, and *sw-4*, and three dominant genes *Sw-5*, *Sw-6*, and *Sw-7*^48,49^. *Sw-5*, originally introgressed in the cultivar ‘Stevens’, is currently the primary source of TSWV resistance in commercial tomato varieties worldwide^50,51^. Domesticated tomato is known to be vulnerable to *Tomato yellow leaf curl virus* (TYLCV) infection (Ji et al., 2007). Five major loci resistant to TYLCV (*Ty-1, Ty-2, Ty-3, Ty-4*, and *Ty-5*) have been introgressed from different wild relatives into tomatoes^51,52^. To date, only one dominant gene, *Sm*, that confers effective resistance to *Stemphylium lycopersici*, a fungus responsible for tomato gray leaf spot disease, was identified in the wild tomato species *S. pimpinellifolium*, and has been used to breed resistant tomato cultivars^53^. Other four plant R-genes have been introgressed from wild tomato species including the *I* (or *I-1*) and *I-2* from *S*. *pimpinellifolium*, and the *I-3* and *I-7* from *Solanum pennellii*, with *I-2*, *I-3*, and *I-7* encoding an NBS-LRR protein and *I-2* and *I-3* conferring resistance to race 2 and race 3 strains of *Fusarium oxysporum* f. sp. lycopersici (FOL), respectively^54,55^. To date, four major dominant genes, *Rx4* and *RxLA1589* in *S. pimpinellifolium*^56–58^, *Xv3* in the unimproved breeding line Hawaii7981^59^, and *RXopJ4* in *S. pennellii*^60^, conferring HR to *Xanthomonas perforans* race T3 have been identified and mapped. Several *Phytophthora infestans* resistance genes (*Rpi*) have been identified from different *Solanum* spp., mainly wild potato species, and also from tomato. Five major *Rpi* genes (Ph-1–Ph-5) were identified in different accessions of *S. pimpinellifolium*, and *Solanum habrochaites*^51^. Efforts to develop *Phytophthora* resistant tomatoes by using individual *Rpi* genes were not successfully achieved^61,62^.

The existence of specific R-genes against a variety of pathogens turns tomato specially interesting to develop genetic studies of plant host-specific resistance mechanisms^41,51,63^.

## Next-generation sequencing applied to study tomato-pathogen interactions

**Fig. 1:**
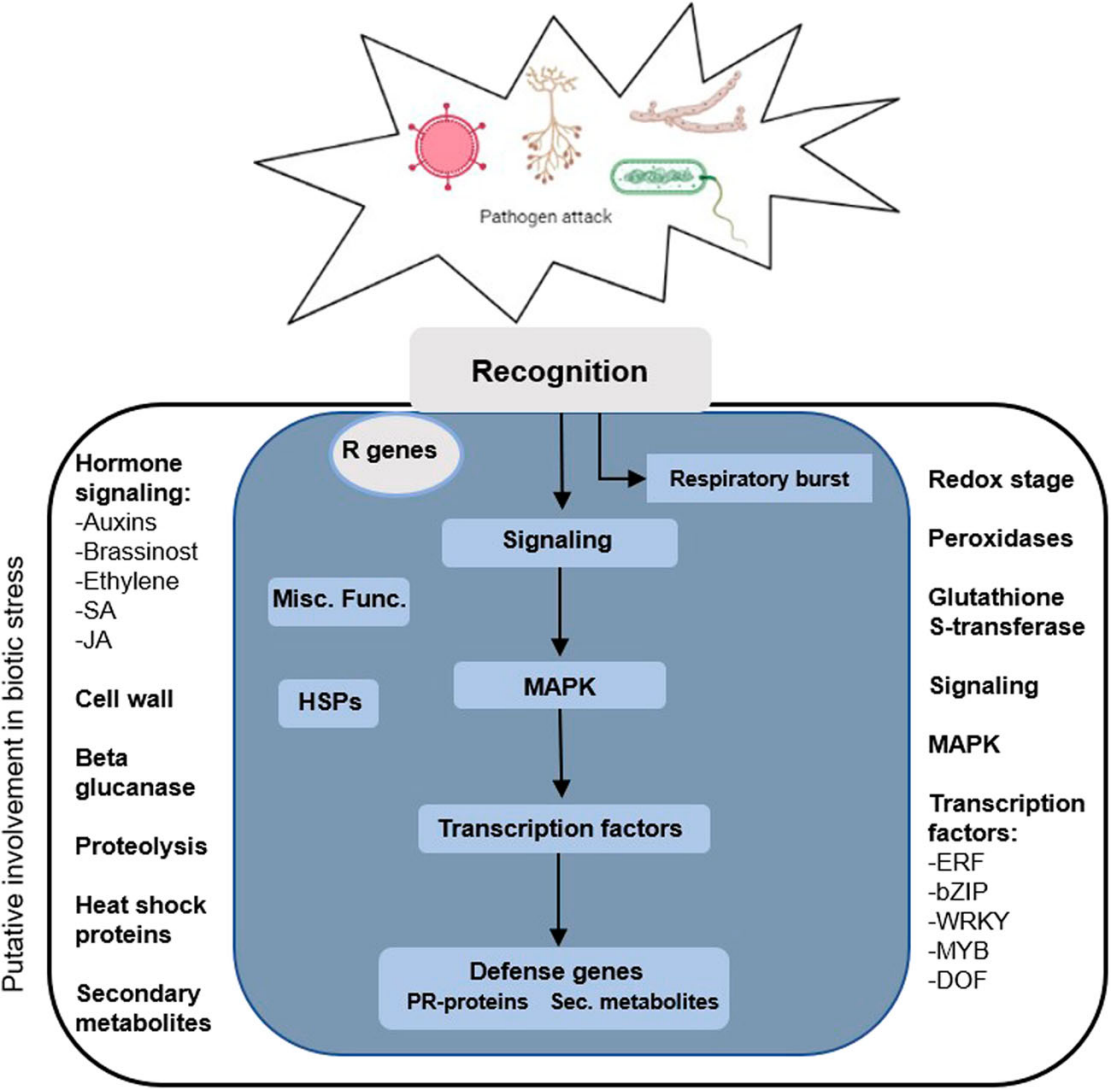
Regulatory overview of the differentially expressed genes involved in plant response to biotic stress. The plant’s reaction to biotic stress involves a few steps: after the initial signal input from the pathogen which is recognized by the related receptors (putative R genes), transcription of the cascade of the plant defense mechanism is triggered, including oxidative stress changes. Inside the cell, signals are transmitted to lead to the production of defense molecules (PR-proteins, heat shock proteins, and secondary metabolites). Genes with an experimental indication of involvement in the biotic stress are gathered on the main panel (colored with blue), while genes and pathways that are putatively involved in the biotic stress pathway are shown on the left and right sides (adapted from^66^).

The development of novel methodologies allows a better understanding of the molecular mechanisms involved in the interaction between the plant and each specific pathogen. Several studies utilizing proteomic and metabolomic techniques have documented the global responses of tomato to infections (^64,65^ and references within). However, it is of fundamental importance to uncover changes in tomato gene expression following infection, that will help to exploit key genes in both resistant and susceptible responses to the pathogens. Hence, the understanding of the plant genes’ network involved in the activation of antipathogenic responses is essential for the development of a molecular toolbox that can estimate the tolerance or resistance to diseases. To obtain an overview of the regulatory pathways induced in plants by pathogen infection, potentially differentially expressed genes (DEGs) are indicated in Fig. 1.

Transcriptome analysis is a very important tool to discover the molecular basis of plant-pathogen interaction globally, allowing dissection of the pattern of pathogen activities and molecular repertoires available for defense responses in host plants. Next-generation sequencing (NGS) technologies, which produce massive quantities of sequencing data, have greatly accelerated research in biological sciences^19,41,67^. Implementation of high-throughput methods, including microarray^68,69^ and high-throughput RNA-seq technology, enable wide transcriptome profiling, which is very helpful in understanding the molecular networks of plant/host-pathogen interactions^70,71^. RNA-seq does not require prior knowledge of genome sequences, allows deep sequencing of the transcriptome understudy, and has been used to obtain transcriptome changes in plant response to disease infection^72,73^. Microarrays are relatively inexpensive compared to RNA-seq, but RNA-seq has many advantages, namely higher gene coverage and increased sensitivity in gene expression monitoring, with more transcripts identified^69^.

Tomato is, as referred above, susceptible to several diseases caused by an array of pathogens. Taking advantage of an RNA-seq approach, transcriptome studies on tomato response to pathogen infection, shed light on the cross-talking among different signaling pathways involving tomato-pathogen interaction.

**Fig. 2:**
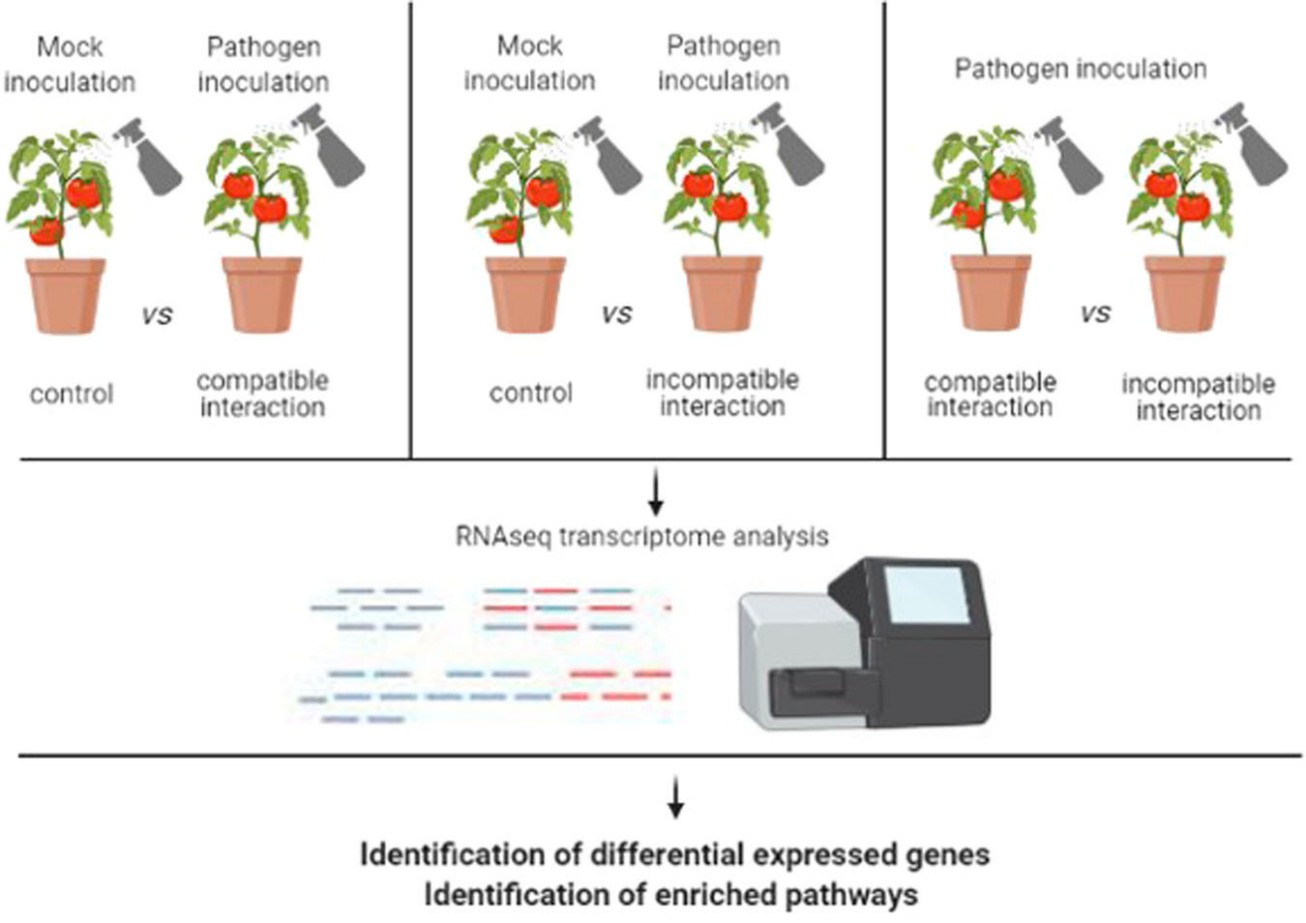
Schematic presentation of the approach followed for transcriptomic studies on tomato response to biotic stress analysis. A: susceptible cultivar *vs*. control; B: resistant cultivar *vs*. control; C: susceptible cultivar *vs*. resistant cultivar. Created with BioRender.com

Bellow, we describe relevant research based on RNA-seq technology to study tomato response to infection by several pathogens and report relevant findings highlighted by the authors. In view of the identification of key functional genes, it is described the application of this technology to tomato infection by several viruses and viroids, fungi, oomycetes, bacteria, and nematodes, responsible for important economic losses. Although the methodology followed by the different authors was quite similar, some of these studies were supported by the important advantage of the use of pathogen-resistant tomato cultivars to the different pathogens (a resistant and a susceptible response were compared), while other studies are based on the comparison of the transcriptomic profile of control *versus* inoculated tomatoes, as susceptible or resistant hosts (with a compatible or incompatible interaction with the pathogen), or still using mild or severe pathogen variants. A schematic presentation of the experimental design used for transcriptomic studies on tomato response to biotic stress analysis is represented in Fig. 2.

It was performed the identification of enriched pathways of the DEGs involved in the response of tomato to infection by an extended number of pathogens, which are indicated in Table 1.

**Table 1:** Identification of enriched pathways of the differentially expressed genes associated with response of tomato to infection by several pathogens, highlighted by the respective authors. Resistant (R) or susceptible (S) cultivars/breeding lines, and the organs infected by the pathogens are indicated.

Pathogen	Cultivars/breeding lines	Infected organs	Pathways of the differential expressed genes	Ref.
*Tomato spotted wilt tospovirus* (TSWV)	Sw-7 (R) Fla. 8059 (S)	Leaves	- Host defense-related genes, transcription factors, protein kinases, phytohormones signaling, cell wall-related genes (comparison between susceptible and resistant response).	^76^
*Tomato yellow leaf curl virus* (TYLCV)	CLN2777A (R) TMXA48-4-0 (S)	Leaves	- Cell wall reorganization, transcriptional regulation, defense response, ubiquitination, metabolite synthesis (upregulated in resistant response).	^52^
*Potato spindle tuber viroid* (PSTVd)^a^	Rutgers (S)	Leaves	- Biosynthesis of secondary metabolites, cysteine and methionine metabolism, ribosome, spliceosome, protein processing in endoplasmic reticulum, proteasome, phagosome, regulation of autophagy, peroxisome, endocytosis, plant-pathogen interaction, plant hormone signal transduction (in a susceptible response).	^69^
*Verticillium dahliae*	Micro-Tom (S)	Roots	- Phenylalanine metabolism and phenylpropanoid biosynthesis, plant hormone signal transduction, plant–pathogen interaction, cysteine and methionine metabolism, protein processing in endoplasmic reticulum, oxidative phosphorylation pathways (in a susceptible response).	^84^
*Fusarium oxysporum*	Motelle (R) Moneymaker (S)	Roots	- Phenylpropanoid biosynthesis, biosynthesis of secondary metabolites, metabolic pathways (in both a resistant and susceptible response). - Plant-pathogen interaction (in a resistant response).	^87^
*Alternaria solani*	Pusa Ruby (S)	Leaves	- Metabolic pathways, biosynthesis of secondary metabolites, plant hormone signal transduction, phenylpropanoid biosynthesis, plant-pathogen interaction (in a susceptible response).	^88^
*Stemphylium lycopersici*	Motelle (R) Moneymaker (S)	Leaves	- Plant–pathogen interaction, plant hormone signal transduction, regulation of autophagy (in a resistant response). - Plant hormone signal transduction, glycerophospholipid metabolism, alpha-linolenic acid metabolism (in a susceptible response).	^91^
*Cladosporium fulvum*	Ontario7816 (R) Moneymaker (S)	Leaves	- Plant hormone signal transduction, plant-pathogen interaction (in a resistant response). - Plant-pathogen interaction, plant hormone signal transduction (among overlapping genes between resistant and susceptible response).	^93^
*Xanthomonas perforans* Race T3	PI114490 (R) OH88119 (S)	Leaves	- Photosynthesis, oxidative phosphorylation, phenyl alanine metabolism, glutathione metabolism, phenylpropanoid biosynthesis, ribosome, plant hormone signal transduction, plant-pathogen interaction (in a resistant response). - Protein processing in endoplasmic reticulum, MAPK signaling pathway, plant hormone signal transduction, oxidative phosphorylation, photosynthesis, ribosome, phenylalanine metabolism, metabolism of xenobiotics by cytochrome P450 (in a susceptible response).	^95^
*Phytophthora parasitica*	LA2093 (R) LA1581 (S)	Leaves	- Mitogen activated protein kinase (MAPK) signaling pathway; plant hormone signal transduction pathway (in both a susceptible and a resistant response).	^94^
*Meloidogyne incognita*	M36 (R) Pusa Ruby (S) Moneymaker (S)	Roots	- Cell wall structure, development, primary and secondary metabolite, and defense signaling pathway (in a susceptible response). - Secondary metabolite and hormone-mediated defense responses (in a resistant response).	^98^

^a^based on microarray analysis.

Resistant (R) or susceptible (S) cultivars/breeding lines, and the organs infected by the pathogens are indicated.

### Tomato-viruses and viroids interactions

RNA-seq technology has already been applied to tomato response to virus infection, such as the one caused by *Tomato spotted wilt virus* (TSWV), one of the most important viruses that infect tomato worldwide. TSWV causes plant stunting and chlorotic or necrotic spots on leaves and fruits, resulting in high yield losses. Its easy transmission through thrips has contributed to the worldwide dispersion with great impacts on agriculture and food security^74,75^. In order to uncover gene networks that are associated with TSWV resistance, Padmanabhan et al.^76^ performed a comparative transcriptome profiling between the resistant *Sw-7* line and its susceptible recurrent parent, upon infection. These authors report a total of 1244 DEGs throughout a disease progression process involving networks of host resistance genes, RNA silencing/antiviral defense genes, and crucial transcriptional and translational regulators. The genes induced in the tomato *Sw-7* line include those involved in callose accumulation, lignin deposition, proteolysis process, transcriptional activation/repression, and phosphorylation. Several categories of genes involved in the *Sw-7* resistance response are indicated in Table 1, with candidate genes including those encoding nucleotide-binding site leucine-rich repeat (NBS-LRR, R-genes) proteins, defense-related proteins, TFs, protein kinases, as well as those related to phytohormone signaling, cell wall, photosynthesis, gene silencing, and microRNA target genes. On contrary, the inoculated susceptible S-line plants experienced significant changes in gene expression related to a variety of general immune system and defense response pathways, including phytohormone synthesis. A total of 27 PR-protein family genes with differential expression between the *Sw-7* line and susceptible S-line were identified.

Other important virus causing severe losses is the *Tomato yellow leaf curl virus* (TYLCV). However, the knowledge of the host plant defense response to TYLCV is very limited. TYLCV causes stunting, leaf curling and yellowing, and flower abscission leading to critical tomato production losses^77^. It is transmitted mainly by the whitefly *Bemisia tabaci* Gennadius (Hemiptera: Aleyrodidae)^78^ which is responsible for its great dissemination worldwide^79^. In order to understand the mechanism involved in tomato defense, Chen et al.^52^ analysed the differential gene expression in response to TYLCV infection in a TYLCV-resistant breeding line and a TYLCV-susceptible breeding line, following an RNA-seq approach. Chen et al.^52^ found that there was a higher proportion of upregulated differentially expressed genes in the tomato resistant line (58.37%) than that in the susceptible line (9.17%). However, the defense responses of these two tomato lines were quite different, with 209 and 807 genes differentially expressed in the resistant and susceptible lines, respectively. In response to TYLCV infection, the authors highlighted upregulated DEGs in the resistant line associated with plant defense response at different levels, such as cell wall formation and reorganization, ethylene response, ubiquitination during plant immune signaling, metabolite synthesis, ranging from the regulation of TFs to the activation of defense genes and to the post-translational modification of proteins that participate in the defense response to pathogen infection. Some sets of defense-related genes encoding for WRKY TFs, R genes, protein kinases, and receptor (-like) kinases that exhibited a dramatic down-regulation in the susceptible line were upregulated or not differentially expressed in the resistant line.

The approach followed to study transcriptional tomato roots response to infection by the *Potato spindle tuber viroid* (PSTVd) viroid was based on the comparison of mild or severe PSTVd variants^69^. Viroids infect higher plants systemically and can cause several disease symptoms similar to those observed during plant viral infection, including stunting, epinasty, chlorosis, and necrosis or malformation of tubers, flowers, and fruits^80^. Góra-Sochacka et al.^69^ verified that 1168 and 1101 genes were differentially regulated during mild and severe infection, respectively. Comparison of both PSTVd infections showed that transcriptional changes induced by the severe variant were stronger than those caused by the mild variant. DEGs identified from RNA-seq related to cell wall organization, modification and degradation were repressed in both infections. Another common enriched category was ‘response to stress’, which includes genes that encode peroxidases, defensins, and MLP-like proteins. The authors highlighted changes in the expression of genes that encode MAPKs, WRKY TFs, CDPKs, NBS-LRRs (R genes), PR-proteins, receptor-like kinases (RLKs), and others from a plant-pathogen pathway, indicating these results the activation of the plant immune response.

### Tomato-fungi interactions

There are several examples of the application of RNA-seq technology to plant response to fungal infection. One of the studies was performed on the vascular wilt pathogen, *Verticillium dahlia*. Vascular wilt diseases caused by soil-borne pathogens are among the most devastating plant diseases worldwide and cause severe reductions in tomato yield and quality^81^. The infection of plants by *V. dahliae* results from penetration of young roots via wounds or cracks that occur at the sites of lateral roots^82,83^. The high economic impact of these diseases, combined with the absence of curative treatments, justifies increased attention on them. The approach followed by Tan et al.^84^ with the study on tomato-*V. dahlia* interaction is based on the comparison of the transcriptomic profile of inoculated tomatoes (a susceptible host, with a compatible interaction with the fungi), with noninoculated tomatoes (control group), aiming to identify key functional genes in susceptible responses and understand the molecular basis of compatible interactions. The authors identify important functional groups responsible for fundamental biological regulation, secondary metabolism, signal transduction, and DEGs assigned to several pathways, mostly associated with phenylpropanoid metabolism and plant–pathogen interaction pathways (Table 1). The majority of the DEGs involved in these two pathways were upregulated, and may be involved in regulating the tomato-*V.dahliae* compatible interaction.

*Fusarium* species are ubiquitous soil-borne pathogens of a wide range of horticultural and food crops which cause destructive vascular wilts, rots, and damping-off diseases^85^. *Fusarium oxysporum* f.sp. *lycopersci* (FOL) is the causal agent of the tomato wilt disease, a worldwide destructive disease of tomato^86^. FOL enters the epidermis of a root, later spreads through the vascular tissue, and inhabits the plant xylem vessels, resulting in vessel clogging, and severe water stress as a result wilt-like symptoms appear^39^. The analysis of the transcriptome of tomato root under FOL infection brought new insights into the tomato wilt disease response. The interaction between FOL and tomato is race-cultivar-specific. Two near-isogenic tomato cultivars susceptible (i-2/i-2) and resistant (I-2/I-2) were recruited to study the interaction between tomato and FOL^87^. FOL treatment had a significant impact on RNA gene expression profile in tomato plants, since DEGs belonging to several different pathways were identified^87^ (Table 1). There were differentially regulated a higher number of pathogen resistance genes in the resistant cultivar than in the susceptible cultivar. These DEGs included, among others, genes encoding WRKY protein, receptor kinase, MYB TF, NBS-ARC protein, Calmodulin-like protein, and MAPK.

Tomato response to infection by the fungi *Alternaria solani* is another example of the application of RNA-seq technology to fungal infections. By studying a susceptible cultivar and comparing inoculated and noninoculated plants, Sarkar et al.^88^ found 5450 DEGs. This analysis also revealed that more genes were upregulated than downregulated. Concerning the exact biological processes that the DEGs may participate during *A. solani* stress, several important pathways were significantly enriched (Table 1). Enrichment analysis highlighted genes associated with photosynthesis, suggesting that plant photosynthesis is affected during infection due to chlorosis and blight progression during the disease. In addition, biosynthesis of secondary metabolites and phenylpropanoids, plant–pathogen interaction, and plant hormone signal transduction pathways were significantly enriched, suggesting substantial regulation of plant hormone signaling and plant defense during the infection.

Gray leaf spot disease caused by *Stemphylium lycopersici* is one of the most destructive diseases in cultivated tomato plants threatening tomato-growing areas worldwide^89^. In the early stages, tomato gray leaf spot disease symptoms appear as brownish-black specks, which later expand to necrotic lesions with gray centers and dark brown borders. As the disease progresses, affected leaves became chlorotic with perforated centers of lesions, that dry and fall^90^. Aiming to analyse the regulatory resistance mechanisms of the resistant tomato cultivar in response to *S. lycopersici*, Yang et al.^91^ inoculated a resistant cultivar and a susceptible cultivar with a virulent *S. lycopersici* isolate and performed a transcriptome analysis. RNA-seq approach revealed that the overall number of DEGs was higher in the resistant than in the susceptible cultivar. Additionally, the number of upregulated genes was greater than the number of downregulated genes in the two tomato cultivars. The functions of DEGs involved in the response to *S. lycopersici* infection and functional classification of DEGs are indicated in Table 1. Functional classification revealed that most DEGs were involved in plant–pathogen interactions, plant hormone signal transduction, regulation of autophagy, glycerophospholipid metabolism, and alpha-linolenic acid metabolism (Table 1)^91^. In total, the most-enriched pathways, ‘Plant–pathogen interaction’ (111 DEGs) and ‘Plant hormone signal transduction’ (78 DEGs) may be the major metabolic pathways involved in the resistant tomato response to *S. lycopersici* infection. Yang et al.^91^ highlighting the DEGs related to disease-resistance pathways that were significantly upregulated in the resistant tomato cultivar.

A comparative transcriptomic analysis was performed in resistant and susceptible tomato cultivars infected with *Cladosporium fulvum*. *C. fulvum* is a nonobligate, abiotrophic pathogenic fungus that infects foliage and occasionally petioles and stems, causing the leaf mold disease^20^. Leaf mold has long been prevalent in many countries and caused serious economic losses, especially under high temperature and high humidity conditions^92^. Zhang et al.^93^ revealed that after pathogen inoculation more DEGs were found in the resistant tomato cultivar than in the susceptible one, especially upregulated genes. Systemic defense response mediated by resistance genes was activated during the early stage of *C. fulvum* infection. Upregulated genes in resistant tomato were primarily associated with defense processes and phytohormone signaling, including salicylic acid (SA) and jasmonic acid (JA) (Table 1).

### Tomato-oomycetes interactions

Oomycetes in the genus *Phytophthora* are responsible for several devastating diseases in tomatoes such as late blight, *Phytophthora* root, crown rot, and buckeye rot. These diseases not only damage tomato crop production but also cause major postharvest losses, threatening the tomato processing industry. *P. parasitica* is mainly known as a root and fruit pathogen of tomato associated with Phytophthora root rot and buckeye rot diseases but leaf infection, stem canker, stem girdling, collar rot, blossom blight, and damping-off of seedlings have also been reported in tomatoes in different parts of the world^20^. To understand the molecular basis of resistance against *P. parasitica*, Azal Naveed and Ali^94^ compared *P. parasitica* resistant and susceptible accessions of the wild relative tomato *S. pimpinellifolium* in response to infection. By comparing inoculated vs. control plants, these authors identified 2657 DEGs in the resistant accession and 3079 DEGs in the susceptible one. Functional annotation of DEGs revealed substantial transcriptional reprogramming of diverse physiological and cellular processes, particularly the biotic stress responses in both resistant and susceptible upon *P. parasitica* treatment. The results revealed that from the 2657 DEGs identified, 1173 genes were differentially expressed exclusively in resistant accession upon *P. parasitica* inoculation. DEGs included core plant defense genes, for example, several protease inhibitors, chitinases, defensin, PR-1, a downy mildew susceptibility factor, all highly induced; on the contrary, several R genes, WRKY TFs, and a powdery mildew susceptibility gene were repressed during the resistance outcome. The functional involvement of DEGs in biological pathways is indicated in Table 1.

### Tomato-bacteria interactions

As for other pathogens, there are examples of the application of RNA-seq technology to tomato response to bacterial infection. Mainly four distinct species of *Xanthomonas* (*X. euvesicatoria*, *X. vesicatoria*, *X. perforans*, and *X. gardneri*) cause bacterial spot, a disease that severely affects marketability of both fresh-market and processed tomato^20^. Based on their virulence on a group of tomato genotypes, the bacteria *Xanthomonas perforans* can be classified into five physiological races T1–T5^20^.

Focusing on the tomato resistance to *X. perforans* race T3, to fully unravel the mechanisms of field resistance and to identify differentially expressed genes during different infection times in tomato, Due et al.^95^ investigated the post-infection transcript dynamics of a field resistant line and of a susceptible line. In the face of a large number of commonly upregulated genes, the authors report to the highly upregulated genes in both resistant and susceptible lines. They observed that marker genes of stress signaling such as PR genes and osmotin-like protein were intensely induced, with genes associated with defense response pathways being significantly upregulated in the resistant line. The top enriched defense-related pathways were planted hormone signal transduction, plant-pathogen interaction, and phenylalanine metabolism. DEGs containing nucleotide-binding site-leucine rich repeat (NBS-LRR, R genes) domain or defense-related WRKY TFs were also identified. Plant-pathogen interaction response usually alters the expressional level of genes associated with photosynthesis^96^, however, RNA-seq data from Du and co-workers showed that race T3 pathogen caused an opposite impact on photosynthesis between resistance and susceptible tomato lines, with the genes involved in photosynthesis pathway being mainly upregulated in the susceptible tomato line (Table 1).

The transcriptome-based studies of tomato resistance to *X. perforans* race T4^97^ followed a similar approach to the one described to the resistance to race T3. These authors identified unique differentially expressed genes in resistance, such as upregulated PR-protein genes specific to this study. On the other hand, a disease-associated R gene was found downregulated in the susceptible line. The accession resistant to race T4 had more DEGs compared to the susceptible line, as happened with race T3 resistance, but the line with a medium level of resistance, had fewer DEGs induced by inoculation of *X. perforans* race T4. Additional DEGs were identified in tomato resistance to *X. perforans* race T4 that had not been reported in the study conducted by Du et al.^95^. Shi and Panthee^97^, refer that a possible reason is that samples analysed were collected in different timepoints, and that the studied varieties and lines had a different genetic background.

### Tomato-nematodes interactions

Root-knot nematodes belong to the genus *Meloidogyne* and are devastating polyphagous endoparasites that parasitize many cultivated plants worldwide, causing important economic losses.

A comprehensive transcriptomic approach was followed to investigate the expression of susceptible and resistant tomato genes in roots at several infection time intervals in both *Meloidogyne incognita* susceptible and resistant lines. RNA-seq data revealed 1827 tomato DEGs during susceptible and 25 tomato DEGs during resistance responses^98^. An alteration in the expression of tomato genes involved in cell wall degradation, cell wall modification, cell wall proteins, and cell wall synthesis was verified during the susceptible response, as well as genes involved in the development, primary and secondary metabolite, and defense signaling pathways^98^. By contrast, during resistance response, no significant alteration was observed in the expression of genes involved in the modulation of cell wall architecture, whereas tomato genes involved in secondary metabolite and hormone-mediated defense responses are indicated^98^ (Table 1). The components of ethylene, abscisic acid, and SA signaling were differentially regulated during both the susceptible and resistance responses^98^. In a different trial, but also studying the transcriptome analysis of four tomato genotypes, (with different levels of susceptibility to the nematode), under *M. incognita* stress, Kulshrestha et al.^99^, verified differential gene expression for chitinase activity, PAL, SAM, BURP, and peroxidases.

## Tomato functional genomics in view of plant breeding

Host resistance is an important component of a sustainable disease management system^17^. It is an environmentally benign method that can be used to replace costly and unsustainable chemical controls, with environmental and human health effects^17,100,101^, and even more relevant with the emergence of resistant pathogen/pest strains^102^. The identification of plant regulatory components involved in protection against pathogens can therefore be of major importance for sustainable plant-disease management, namely the ones relying on the plant innate immune mechanisms.

Identification of genetic sources of resistance against pathogens can serve as a valuable resource for developing resistant crops. The approach following the transcriptomic analysis should further characterize the functions of the selected candidate DEGs involved in plant-pathogen interactions and elucidate the role of the genes involved in susceptibility and/or resistance. This will help to determine the detailed regulatory mechanisms of plant diseases and develop new strategies for controlling plant pathogens. The identified DEGs from the research described above point to the involvement of different pathways. We highlight the pathways ‘plant hormone signal transduction’, ‘plant-pathogen interaction’, and ‘biosynthesis of secondary metabolites’ as common to tomato response across several pathogens (Table 1). Our study report genes encoding nucleotide-binding NBS-LRR proteins and TFs, commonly identified in tomato response to pathogens. As far as the availability of data allows, it is no possible the identification of a pattern of specific DEGs associated with the type of pathogen (viruses *vs* fungi *vs* oomycetes *vs* bacteria *vs* nematodes), as well as soil-borne pathogens and pathogens that affect specific tomato organs. Probably this could be overcome with the increase of the number of tomato RNA-seq studies in response to a broader range of pathogens.

Identified candidate genes might then be tested in strategies involving gene knockout or overexpression, that will facilitate breeding and genetic engineering efforts to incorporate a new source of resistance in tomato.

Some of the RNA-seq research already allowed the identification of key genes involved in tomato response to a pathogen that were used in functional studies. Padmanabhan and co-workers^76^ identified a *PR-5* gene possibly involved in tomato resistance against TSW. The functional characterization revealed that *PR-5* overexpressed plants conferred enhanced resistance, resulting in a delay in virus accumulation and symptom expression^76^. Also, Chen et al^52^. identified a gene encoding an NBS-LRR protein (Solyc05g009760), whose silencing in the resistant background led to increased TYLCV accumulation. RNA-seq was also applied to the interaction of tomato with *Pseudomonas syringae* pv*. tomato* to identify genes whose expression changes specifically during pattern-triggered or effector-triggered immunity. Virus-induced gene silencing of ETI-specific genes identified *Epk1*, which encodes a predicted protein kinase from a family previously unknown to be involved in immunity. Knocked-down expression of *Epk1* compromised ETI triggered by three bacterial effectors but not by effectors from non-bacterial pathogens^103^.

## Conclusion

The availability of NGS techniques opens the possibility of characterizing tomato transcriptomic responses to different pathogen challenges. Knowledge acquired through RNA-seq yields new insights into the molecular mechanism of tomato response to infections. Overall, we can find similar or specific sets of genes activated in different tomato pathosystems, and there were identified DEGs that belong to different pathways. The great amount of data contributes for the identification of key genes, that are valuable resources to study the tomato-pathogen interaction, and adopt a strategy looking for tomato breeding. Functional genomics plays undoubtedly a key role in our current understanding of the defense response in tomato, opening challenges and opportunities for the future.
